# A stalk fluid forming above the transition from the lamellar to the rhombohedral phase of lipid membranes

**DOI:** 10.1007/s00249-020-01493-2

**Published:** 2021-02-15

**Authors:** Max Scheu, Karlo Komorowski, Chen Shen, Tim Salditt

**Affiliations:** 1 Institute for X-ray Physics, Friedrich-Hund-Platz 1, 37073 Göttingen, Germany; 2 DESY Photon Science, Notkestr.85, 22607 Hamburg, Germany

**Keywords:** Fusion stalk, Lipid membranes, Grazing-incidence X-ray diffraction, Smectic elasticity

## Abstract

In this work, we present evidence for the formation of transient stalks in aligned multilamellar stacks of lipid membranes. Just above the phase transition from the fluid ($L_\alpha$) lamellar phase to the rhombohedral phase (R), where lipid stalks crystallize on a super-lattice within the lipid bilayer stack, we observe a characteristic scattering pattern, which can be attributed to a correlated fluid of transient stalks. Excess (off-axis) diffuse scattering with a broad modulation around the position which later transforms to a sharp peak of the rhombohedral lattice, gives evidence for the stalk fluid forming as a pre-critical effect, reminiscent of critical phenomena in the vicinity of second-order phase transitions. Using high-resolution off-specular X-ray scattering and lineshape analysis we show that this pre-critical regime is accompanied by an anomalous elasticity behavior of the membrane stack, in particular an increase in inter-bilayer compressibility, i.e., a decrease in the compression modulus.

## Introduction

**Fig. 1:**
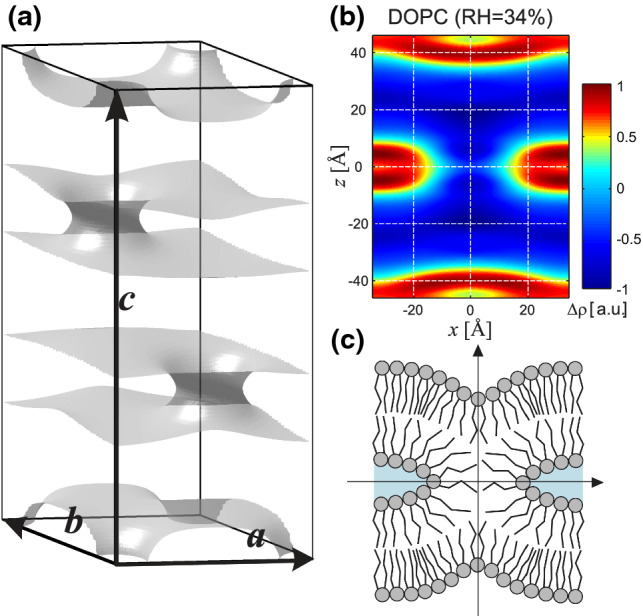
Illustration of the stalk phase. **a)** Three-dimensional arrangement of stalks visualized as iso-lines of the electron density, and nonprimitive hexagonal unit cell with basis vectors, from Aeffner (2011). **b)** Normalized electron density of a stalk in a pure 18:1 ($\varDelta$9-Cis) PC (DOPC) system at 34 $\%$ RH, reconstructed from the reflections of the rhombohedral phase, from Aeffner et al. (2012). **c)** Schematic illustration showing the stalk cross-section, from Aeffner et al. (2012)

Biological membranes are nature’s ubiquitous interfaces, built from a robust lipid bilayer architecture which prevents unwanted rupture, adhesion or fusion, based on their elastic properties and inter-membrane forces. In special situations, however, membranes have to fuse in a controlled process, for example in vesicle trafficking, including release of neurotransmitters via exocytosis. To achieve fusion, energy has to be provided to bring the bilayers together and perform work against the hydration repulsion (Schneck et al. 2012). This is accomplished by fusiogenic proteins such as SNARE proteins which mediate fusion in a vast class of vesicle processes (Jahn et al. 2003). The required energy to force the bilayers further together is released by a zippering process between different interacting SNARE proteins anchored in the two opposing membranes (van den Bogaart et al. 2010), resulting in a coiled four-helix bundle (Sutton et al. 1998). In response to local dehydration and mechanical stresses, a so-called stalk structure forms, consisting of a fused proximal monolayer with a highly curved hour-glass shape and an approximately planar and still separated pair of distal monolayers, see Fig. 1. The stalk eventually elongates to a so-called hemifusion diaphragm, which finally ruptures forming a fusion pore. Different roles of the SNARE transmembrane domains in this and related scenarios are reviewed in Kweon et al. (2017).

In 2009, T. Südhof, R. Schekman and J. Rothman have been awarded the Nobel prize for physiology for their discoveries of the machinery regulating vesicle trafficking and in particular the vesicle fusion pathway (Sudhof and Rothman 2009). With ‘machinery’ one typically means proteins and their genes. To understand how they function, structural biology has developed powerful crystallographic tools, providing an atomistic understanding, for example of the four-helix SNARE bundle. For the lipids, however, which actually have to carry out the fusion process, no comparable structural biology approach was available. The lipid stalk structure, first postulated about 30  years ago Kozlov and Markin (1983), was therefore initially investigated only by analytical theory or numerical simulations Chernomordik and Kozlov (2005, 2008), without direct experimental verification. This is not surprising in view of the sub-nanometer resolution required as well as the dynamic nature of stalk formation and transformation, beyond the capabilities of microscopy or protein crystallography. In 2002 finally, Yang and Huang proposed a *lipid-centered* approach to study fusion based on dehydrating lipid membrane stacks Yang and Huang (2002, 2003), Yang et al. (2003). They discovered that stalks can form in equilibrium lipid mesophases, where their structures can be observed and studied in detail by advanced X-ray diffraction; see Fig. 1. Due to the crystallographic symmetry of the lattice, the phase is correctly denoted as a rhombohedral phase, but can more easily be visualized as a stack of membranes with a two-dimensional (2d) hexagonal arrangement of stalks in each plane and a vertical *ABC* stacking. In this work, we will denote the stalk-forming rhombohedral phase synonymously by *stalk phase*. The lipid molecules themselves remain in a fluid phase and conformation (Weinhausen et al. 2012), notwithstanding the long-range order of crystallized stalks.

The stalk phase as a *lipid-only* model system can also be used as a structural fusion assay to classify lipid mixtures in terms of the energy required to form a stalk (Aeffner et al. 2012). The action of fusiogenic proteins which induce a local dehydration, is mimicked by lowering the relative humidity (RH) of the air surrounding a lipid bilayer stack and the corresponding removal of water from the inter-bilayer space. *RH* control can be established, e.g., by mixing two streams of dry and moist nitrogen gas inside the sample chamber using mass flow controllers Aeffner et al. (2009). In our group we used this structural fusion assay to obtain high-resolution reconstructions of stalks Aeffner et al. (2012) and to search for fusion promoting lipid mixtures Khattari et al. (2015), i.e., lipids which undergo the transition to the stalk phase at higher RH, as reviewed in Salditt and Aeffner (2016). We have also undertaken first attempts to incorporate fusiogenic proteins into the stack and to study their effect of stalk formation Xu et al. (2018). An interesting result of Aeffner et al. (2012) was the fact that all mixtures of unsaturated lipids investigated exhibited the lamellar-to-rhombohedral ($L\longrightarrow R$) phase transition, when reaching a critical distance of the water layer $d_{\text {w}}=(9.0\pm 0.5)\,\mathring{\text {A}}$. Here, the inter-bilayer spacing $d_{\text {w}}$ is defined as the distance between adjacent electron density maxima, across the water layer separating adjacent bilayers, as measured in the electron density profile $\rho (z)$ reconstructed from the lamellar diffraction pattern. This finding underlines the importance of the spacing between membranes, which can vary not only on average, as induced by RH, but also locally as induced by proteins or even thermal fluctuation.

One central limitation of this structural fusion assay is given by the fact that stalks are ’arrested’ in the crystalline super-lattice within the fluid membranes, similar to proteins in a crystal, while stalk formation during membrane fusion is intrinsically dynamic. In this work, we present a first step towards the investigation of transient stalk formation. Using high resolution off-specular X-ray scattering from a stack of aligned lipid membranes, i.e., the structural fusion assay as used before in Aeffner et al. (2012), Xu (2017), we now turn to an observation of a characteristic scattering pattern emerging just above the lamellar-to-rhombohedral transition. The diffuse Bragg sheets bend at high $q_\parallel$ into a ’banana’ shape, oriented towards the position where a strong off-axis peak develops when the stalks crystallize in the rhombohedral phase. In a system of pure 1,2-dioleoyl-sn-glycero-3-phosphocholine (DOPC, 18:1 ($\varDelta$9-Cis) PC) lipid membranes, this effect is most pronounced around the first and the fourth Bragg sheet, and can be observed between about 48 % RH and about 40 % RH, at which point the transition to the stalk phase takes place. The intensity of this signal increases with decreasing RH. Due to a behavior which is reminiscent of critical phenomena near a second-order phase transition, we denote this regime as the *pre-critical regime*. As we detail below, we can associate this scattering pattern with fluid, transiently forming stalks before they crystallize at the lamellar-to-rhombohedral transition. Importantly, we show that this pre-critical regime is accompanied by an anomalous elasticity behavior of the membrane stack, in particular an increase in inter-bilayer compressibility, i.e., a decrease in the compression modulus. Finally, we provide an interpretation of these two main findings, the pre-critical anomalous off-axis scattering and the anomalous increase in compressibility.

## Materials and methods

### Sample preparation

1,2-Dioleoyl-sn-glycero-3-phosphocholine (DOPC, 18:1 ($\varDelta$9-Cis) PC) was obtained from Avanti Polar Lipids (Alabaster, AL, USA) and used without further purification. Aligned multilamellar films were prepared by spreading from organic solvent, following the method of Seul and Sammon Seul and Sammon (1990), and earlier work of our group, as reviewed in Salditt et al. (2019). A stock solution was prepared, from which different subsequent solutions were generated. Samples reported here were prepared from a solution of 10 mg/ml DOPC in ethanol, by spreading a volume of 200 $\mu$l (per wafer) onto well leveled and cleaned (ultrasonic bath: 2$\times$ methanol, 2$\times$ ultra-pure water) silicon wafers. The wafers were cut to a size of $2.5 \times 1$ cm$^2$. The surfaces of the wafers were rendered hydrophilic through plasma cleaning (Harrick Plasma, Ithaca, NY, USA), such that the multilamellar film is expected to start by nucleating a bilayer (instead of a monolayer). To remove residual solvent, samples were incubated in a vacuum oven over night, and subsequently equilibrated in a humidity chamber before the measurements. Taking into account the headgroup area of DOPC of $68.9 \pm 0.1 \, \mathring{\text {A}}\,^2$ (Levine et al. 2014), the molar mass of 785.6 g/mol, the average number of bilayers is 4425. The home-built measuring chamber was equipped with a PID RH control to ensure a constant relative humidity (RH) $\pm 0.15\,\%$; see Aeffner et al. (2009).

### Grazing-incident small-angle X-ray scattering

**Fig. 2:**
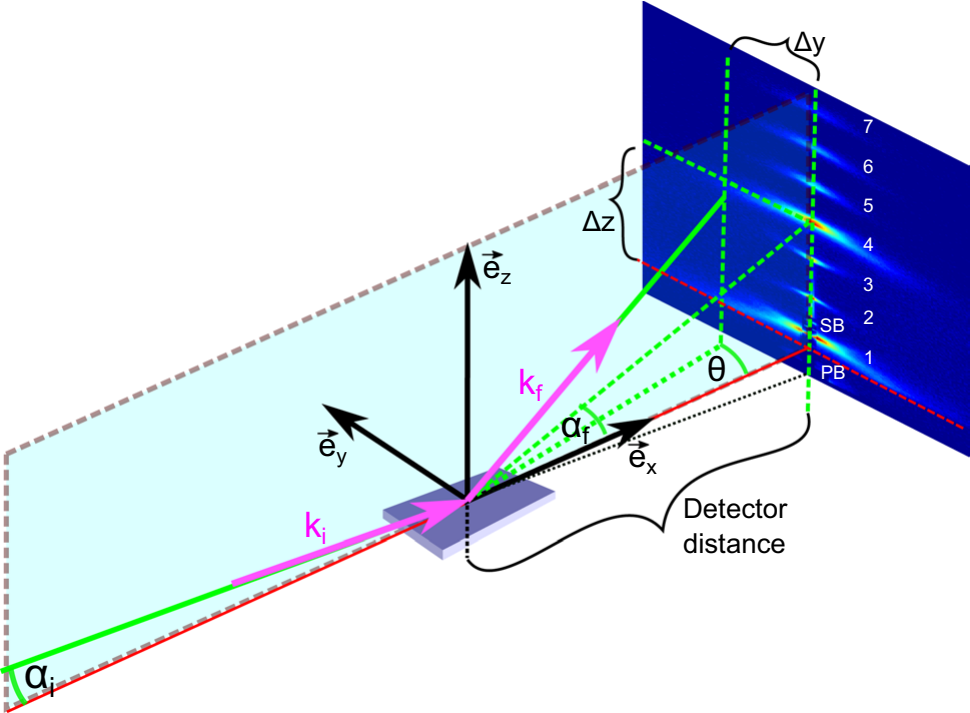
Sketch of the GISAXS geometry used at P08, Petra III/DESY Seeck et al. (2012). The sample horizon at $\alpha _f = 0$ is indicated by the red line. The distance from the center of the sample to the detector plane was 91.55 cm and the angle of incidence was $\alpha _i = 0.4^\circ$. The detector image shows the GISAXS signal obtained from a DOPC multi-bilayer at 42 $\%$ RH, prior to the transition from the lamellar to the rhombohedral phase at 41 $\%$ RH. The first seven Bragg-sheets (diffuse, off-specular signal) can be observed. At fixed angle of incidence, only a single point on the detector contains the specular signal, namely at $\alpha _f = \alpha _i$, and $\theta =0$. The lineshape of the Bragg sheets contains information on the elasticity moduli, accounting for bending and compressional modes

Experiments were carried out at beamline P08 of the PETRA III storage ring (DESY, Hamburg) at 25 keV photon energy (Si(111) monochromator). Note that at this relatively high photon energy, photo-absorption is reduced, and beam damage by the highly brilliant (but unfocused) synchrotron beam, as well as heating of the sample, can be prevented. To this end, scans were repeated several times to check for reproducibility. Further, samples were shifted laterally during longer recordings to spread the dose over the entire surface. The humidity chamber was mounted onto the fully motorized sample stage of the six-circle diffractometer (Kohzu), and the PID control of the RH was integrated into the P08 beamline control software SPOCK via a TANGO server. Humidity was controlled by mass flow control of two mixed nitrogen flows, one fully saturated with vapor pressure, and one dried by passage through silica gel. Both flows were operated at 1.5 bar. The grazing-incidence small-angle X-ray scattering (GISAXS) signal was recorded after careful alignment of all angles and translations by a single-photon counting multi-pixel detector with 172 $\times$ 172 $\upmu \mathrm {m}^{2}$ pixel size and 487 $\times$ 195 pixels (Pilatus 100 k, Dectris), mounted on the diffractometer arm at a distance of 915.5 mm behind the sample. For the data shown here, the angle of incidence was kept constant at $\alpha _i=0.4^\circ$. In order to achieve high signal-to-noise ratio in the GISAXS pattern, 10 diffraction patterns of 10 s exposure time each were recorded for every RH value. This allowed both for a verification that no beam damage or sample drift was present, as well as for improved counting statistics when adding the patterns up. A schematic description of the experimental setup is shown in Fig. 2, optimized to record the off-specular signal as function of momentum transfer parallel $q_\parallel$ and perpendicular $q_z$ to the plane of the bilayers. The data analysis was carried out using Matlab scripts which account for the intensity corrections due to polarization and absorption and transformations from pixel to momentum transfer coordinates (Aeffner et al. 2012; Weinhausen et al. 2012). The script uses the Matlab curve-fitting toolbox to rebin the data to a rectangular matrix with equidistant points in reciprocal space coordinates using an inverse transformation. The resulting data are then analyzed with respect to the smectic structure factor S($q_z$, $q_{||}$); see next section.

### Smectic elasticity coefficients

For notational clarity, we briefly summarize the main results and equations of the smectic elasticity theory used. The thermal fluctuations of an multilamellar membrane stack, oriented in the *xy*-plane, are governed by the free energy density (Hamiltonian) which in linear approximation is written for smectic liquid crystals as De Gennes (1990), Constantin et al. (2003), Lei et al. (1995)

1 $$\begin{aligned} \begin{aligned} H&= \frac{1}{2} \sum _i \int dx dy \left( \kappa ((\partial _x^2 + \partial _y^2 ) u_i(x,y) )^2 \right. \\&\quad \left. + \frac{B}{d} (u_{i+1}(x,y)- u_i(x,y))^2 \right) ~, \end{aligned} \end{aligned}$$

where $u_i$ is the displacement of the *i*th bilayer from its equilibrium (1d lattice) position. The first (Helfrich) term penalizes curvature with the bending modulus $\kappa$, the second term penalizes compression/decompression in a harmonic inter-bilayer potential, as governed by the volume compressibility modulus *B*. Equivalently, a bulk bending modulus $K=\kappa /d$ can also be defined. Note that an additional surface tension term can be neglected for fully hydrated membranes. Spontaneous curvature is assumed to be zero, in view of bilayer symmetry. From *H* and the relevant boundary conditions, the height difference functions $g_{ij}(r) = <(u_i(x,y)- u_j(x',y'))^2>$ can be computed, which by symmetry only depend on the lateral distance $r:=\sqrt{((x-x')^2+(y-y')^2)}$ and the bilayer indices *i* and *j* Constantin et al. (2003). From $g_{ij}(r)$ and the boundary conditions, one can in turn compute the structure factor $S(q_\parallel ,q_z)$ (Salditt et al. 1995; Salditt 2005; Constantin et al. 2003). The functional form of both $g_{ij}(r)$ and $S(q_\parallel ,q_z)$ is governed by the two parameters *B* and $\kappa$, or equivalently by two commonly used ’lineshape’ parameters, namely the *de Gennes* parameter $\lambda$ and the *Caillé* parameter $\eta$. In fact, the ratio of the bending to the compression modulus defines a fundamental length scale of a smectic system, the *de Gennes* parameter or smectic length scale

2 $$\begin{aligned} \lambda = \sqrt{\frac{\kappa }{Bd}} ~. \end{aligned}$$

Further, the unitless Caillé parameter can be defined as

3 $$\begin{aligned} \eta =\frac{\pi k_B T}{2d^2\sqrt{\kappa B/d}} ~. \end{aligned}$$

**Fig. 3:**
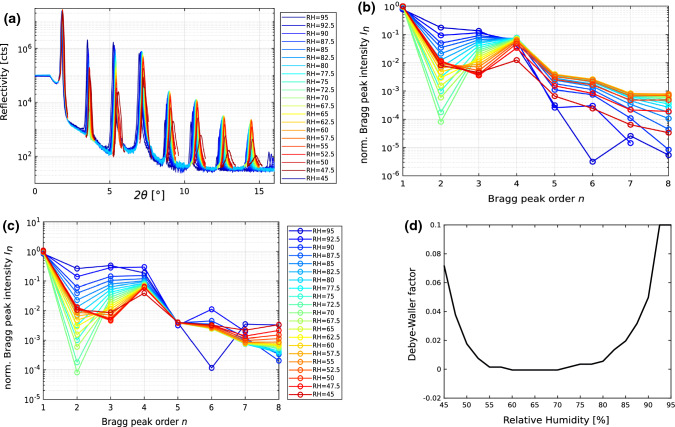
Debye–Waller (DW) factor of specular Bragg peaks. **a** X-ray reflectivity curves as a function of scattering angle $2\theta$ (twice the angle of incidence), data from Aeffner (2011), Aeffner et al. (2012). The different *RH* curves are color coded, as indicated. For intermediate *RH* values (red and yellow curves), the DW factor is small, while it increases both for high humidity, as well as towards the phase transition to the stalk phase. **b** Decay of Bragg peak intensities, after instrumental corrections (Lorentz, polarization, absorption, and illumination factors). In particular, the high order peaks are very sensitive to the DW factor. **c** Corrected Bragg peak intensities, after division by the DW-factor, determined by minimizing the relative deviation of the high order peaks ($n=5$ to $n=8$) with respect to the reference curve $RH_{r}=65\%$, where fluctuations are lowest. **d** Corresponding DW-factor (with respect to $RH_{r}=65\%$) as a function of *RH*. The increase of the DW-factor at low *RH* towards the phase transition reflects the corresponding increase of fluctuations with rms-amplitude $\sigma$ as well as correspondingly $\eta \propto \sigma ^2$; see main text for details

The definition of $\lambda$ and $\eta$ is particularly helpful for scattering analysis, since they can under some approximations directly be inferred from the experimental lineshape, without a full fit to $S(q_\parallel ,q_z)$, which is numerically much more involved. From the determined values of $\lambda$ and $\eta$, for which typically fitting of 1d curves instead of 2d scattering distributions is sufficient, one can then obtain *B* and $\kappa$ in a straightforward manner, i.e.,

4 $$\begin{aligned} \kappa = \frac{\lambda \pi k_B T}{2d \eta} ~, \end{aligned}$$

and

5 $$\begin{aligned} B = \frac{\kappa }{\lambda ^2 d} ~, \end{aligned}$$

with *d* the membrane periodicity (obtained from Bragg peak spacing), $k_B = 1.381 \cdot 10^{-23} ~ \mathrm {J/K}$ the Boltzmann constant, and *T* the temperature. The *Caillé* parameter $\eta$ can be easily extracted from the scattering data for strongly undulating systems of bulk smectic phases. In these cases, the lineshape of the Bragg peaks exhibits strong tails along $q_z$ which are governed by an algebraic decay with exponents given by $\eta$. Contrarily, for oriented stacks on a solid surface, fluctuations are suppressed by the boundary condition and it is not easily possible to extract $\eta$ from a power law tail of a Bragg peak. Full q-range fits, on the other hand, require a lengthy and involved numerical calculation (Salditt 2005; Constantin et al. 2003). In this work, which is primarily concerned with relative changes of the elasticity constants in the vicinity of the phase transition to the stalk phase, we therefore follow a different approach. We determine the de Gennes parameter from the measured full width at half maximum (FWHM) of the peak profile along $q_z$ (see Fig.  3), as a function $q_\parallel$, following the law derived within linear smectic elasticity theory $FWHM = 2\lambda q_\parallel ^2$ (Salditt 2005; Schneck et al. 2011). Taking into account the convolution with an experimental resolution limit, the expression becomes

6 $$\begin{aligned} FWHM = \sqrt{(2\lambda )^2 q_{||}^4 + \varDelta _{res}^2} ~ , \end{aligned}$$

which is the form used for fitting, with $\lambda$ and the resolution $\varDelta _{res}$ as free fit parameters. However, the prefactor in the quadratic relation in Eq. 6 is expected to shift systematically for higher *n*, since the expression requires a linearization for low $q_z \sigma$, where $\sigma$ is the fluctuation amplitude which becomes problematic for higher order Bragg sheets. We therefore first fit an effective prefactor $\lambda ^*_n$ for each Bragg order *n*, and then inspect how the values depend on *n* to approximate $\lambda$. Next to this determination of $\lambda (RH)$ we then determine $\eta (RH)$ from the Debye–Waller (DW) factor $\exp (-\sigma ^2 q_z^2)$ of specular X-ray reflectivity (XRR) measurements, i.e., from the decay of the Bragg peak intensities $I_n$ with lamellar order *n*. Here, $\sigma$ is the rms-fluctuation amplitude averaged over the stack. Finally, the compression modulus *B*(*RH*) and the bending modulus $\kappa (RH)$ will be determined as a function of the relative humidity (*RH*), given the measured $\lambda (RH)$ and $\eta (RH)$.

### X-ray reflectivity and determination of $\eta$

Dehydration of a mulitlamellar stack of membranes strongly affects the thermal fluctuation amplitude and equivalently the Caillé parameter $\eta \propto \sigma ^2$. This is readily inferred from visual inspection of XRR curves of DOPC, where the transition from full to partial hydration reduces the Bragg peak decay, before the decay gets more pronounced again close to the transition to the stalk phase. For the $\eta (RH)$-analysis, which will be based on this effect, we use previously presented XRR measurements, which have been previously measured in our laboratory, as precisely described in Aeffner (2011), Aeffner et al. (2012). Figure  3 therefore only briefly recapitulates the XRR data for DOPC, and explains the new analysis workflow, resulting in $DW(RH)/\eta (RH)$ curves.

In order to develop the analysis scheme, we first have to make the relationship $\eta \propto \sigma ^2$ precise, i.e., include the prefactors. We recall that for solid-supported lipid multilayers, $\sigma ^2$ is obtained from the height–height correlation function evaluated for same lateral distance $r=0$ and for the same vertical position $z=z'$ according to Eq. (15) of Constantin et al. (2003)

7 $$\begin{aligned} \begin{aligned} C(r=0,z,z')&= \eta \left( \frac{d}{\pi }\right) ^2 \sum _{n=1}^{N} \frac{1}{2n-1} \\&\quad \sin \left( \frac{2n-1}{2} \pi \frac{z}{L} \right) \sin \left( \frac{2n-1}{2} \pi \frac{z'}{L} \right) \, , \end{aligned} \end{aligned}$$

where $L=N d$ is the total thickness of the stack. Evaluating the sum, and averaging over the stack, for the given experimental number of bilayers $N=1300$, we get the prefactor as

8 $$\begin{aligned} \sigma ^2 = 2.3 ~\eta ~\left( \frac{d}{\pi }\right) ^2 ~. \end{aligned}$$

Hence, if we knew $\eta$ for each *RH*, we could correct $I_n$ by multiplication with $\exp (n^2 DW)$ with $DW= 2.3 \times (4\eta )$, such that the decay of the Bragg peaks would no longer vary with *RH*. After correcting the intensities for illumination, Lorentz factor, polarization and absorption, as described in Aeffner (2011), this final correction would result in lamellar structure factors $F_{00l}$, from which the electron density profile $\rho (z)$ could be computed based on Fourier synthesis *without* the convolutional effects of undulations. However, here we are not interested in the bilayer profile $\rho (z)$, but in determining the fluctuation amplitude from analysis of the $I_n$ decay. The idea is to find *DW*(*RH*), such that the relative deviation $\sum _n (I_n(RH) -I_n(RH_{r})^2/I_n(RH_{r}^2)$ averaged over a range of *n* is minimized. Here $RH_{r}$ denotes a reference point chosen with known or assumed thermal fluctuation amplitude as a reference. More precisely, $RH_{r}$ is chosen such that the fluctuation amplitude is minimal. From inspection of the XRR curves and $I_n$, the smallest *DW*-factor for DOPC is around $RH_r=65\%$. In fact, the reference value $\sigma _r^2$ and $\eta _r$ at this point is probably dominated by static and not by thermal effects. Independent of this assumption, we can now compute the curve *DW*(*RH*) and $\eta (EH)$ with only a single free parameter $\eta _r$. A posteriori comparison to resulting values for the bending stiffness $\kappa$ leads us to postulate $\eta _r=0.002$, and to keep $\eta _{r,min}=0.0015$, $\eta _{r,max}=0.0025$ as a plausible error interval.

## Results and discussion

The experimentally new contribution of this work is the measurement of $\lambda (RH)$ from a direct analysis of the diffuse reflectivity recorded in this work, notably the Bragg sheet broadening by a simple one-dimensional fit, as detailed below. This behavior is then interpreted in the next section along with $\eta (RH)$ in terms of smectic elasticity near the phase transition.

**Fig. 4:**
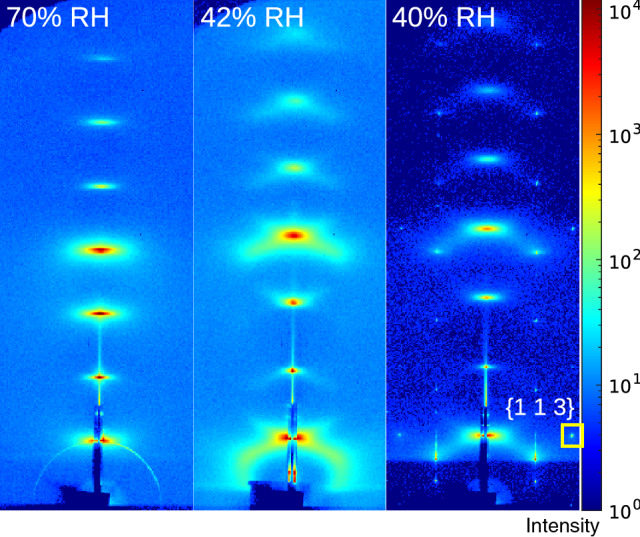
GISAXS patterns of multilamellar stack of DOPC bilayers, at 70 (left), 42 (center), and 40 (right) $\%$ RH, corresponding to (left) the lamellar, and (right) the rhombohedral phase, as well as (center) a regime of anomalous diffuse scattering occurring above the phase transition in a pre-critical regime. On the right, the membrane stack has already entered the rhombohedral phase, as evidenced by the additional off-axis peaks, including notably the off-axis peaks with Miller indices (hexagonal unit cell): (0,1,2), (0,1,5), (0,1,8), and (0,1,11), which are particularly strong Aeffner et al. (2012). The (1,1,3) peak, in particular, is characteristic for the rhombohedral phase and can be used to distinguish it from an inverted hexagonal phase

Figure 4 shows the GISAXS patterns of DOPC multilamellar stack for 70$\%$, 42$\%$, and 40$\%$ RH, representing the fluid $L_\alpha$ phase (lamellar phase), as well as the regime just above the phase transition and the stalk-forming phase (rhombohedral phase). All diffraction patterns have been recorded for the same sample by successively reducing the RH. The angle of incidence was kept constant at $\alpha _i = 0.4^\circ$, such that the specular peak is always located in between the first and second Bragg sheet. It is important to note that the lamellar Bragg sheets recorded in GISAXS geometry represent a purely off-specular (diffuse) signal, different from the lamellar Bragg peaks probed in a reflectivity scan at $q_\parallel =0$. While the latter results only from the lamellar structure, the Bragg sheets arise from the interplay of thermal fluctuations and lamellar order, i.e., require breaking of lateral translation symmetry, notably by membrane undulations. For a perfect stack and for $\lambda \rightarrow \infty$ (or for $T\rightarrow 0$), the Bragg sheets would vanish. In this work, we exploit the characteristic lineshape of the Bragg sheets to infer information about elasticity properties, and in particular about an anomalous elastic behavior just above the transition to the stalk phase. In Fig. 3 this is evidenced by the set of sharp off-axis reflections reflecting the long-range order between the stalks. Importantly, and notwithstanding long-range order of the stalks, the bilayer stack remains fluid with respect to lipid tail ordering and diffusion (Aeffner et al. 2012; Weinhausen et al. 2012).

For DOPC, the transition to the rhombohedral phase occurs at around 41 $\%$ RH (Aeffner 2011). Above the phase transition, the pre-critical regime of increased off-specular intensity is observed, as exemplified by the GISAXS pattern for 42 % RH. The Bragg sheet bends at high $q_\parallel$ into a ’banana’ shape, oriented towards the position where a strong off-axis peak develops when the stalks crystallize in the rhombohedral phase. This is most pronounced around the first and the fourth Bragg sheet, and can be already observed at 48 % RH (not shown). The intensity of this signal increases with decreasing RH, and vanishes again once the transition has been reached, and the stalk phase has formed. In the following and in further detail in Sect. 4, we associate this scattering pattern with fluid, transiently forming stalks before they crystallize at the lamellar-to-rhombohedral transition. Next, we investigate the (smectic) elasticity coefficients of the membrane stack, and will in particular demonstrate that the inter-bilayer compressibility shows an anomalous increase in the pre-critical regime, i.e., a decrease in the compression modulus. In order to determine the elastic coefficients which govern the thermal fluctuations at mesoscopic length scales, and hence the diffraction profiles at small lateral momentum transfer $q_\parallel$, we must inspect the scattering close to the vertical axis ($q_\parallel =0$), i.e., in the region where the Bragg sheets are not yet curved, and measure the Bragg sheet width increase, which is governed by the de Gennes length $\lambda$.

### Lineshape analysis of the diffuse scattering

The determination of the de Gennes parameter $\lambda$ requires least-square fitting of the Bragg sheet width along $q_z$ as a function of $q_\parallel$. According to Eq. 6), the resulting FWHM increases quadratically with $q_\parallel$ with the coefficient given by $\lambda$. To this end, intensity line profiles along $q_z$ were computed from the two-dimensional intensity distributions, and analyzed by batch fits to a Gaussian model. The fit range was adjusted individually to ensure that the parameter values were not falsified. In the pre-critical regime, in particular, the additional critical diffuse scattering associated with stalk formation can also affect the background of the Bragg sheets. This sometimes required an adaptation of the fit range to a smaller range of $q_\parallel$.

**Fig. 5:**
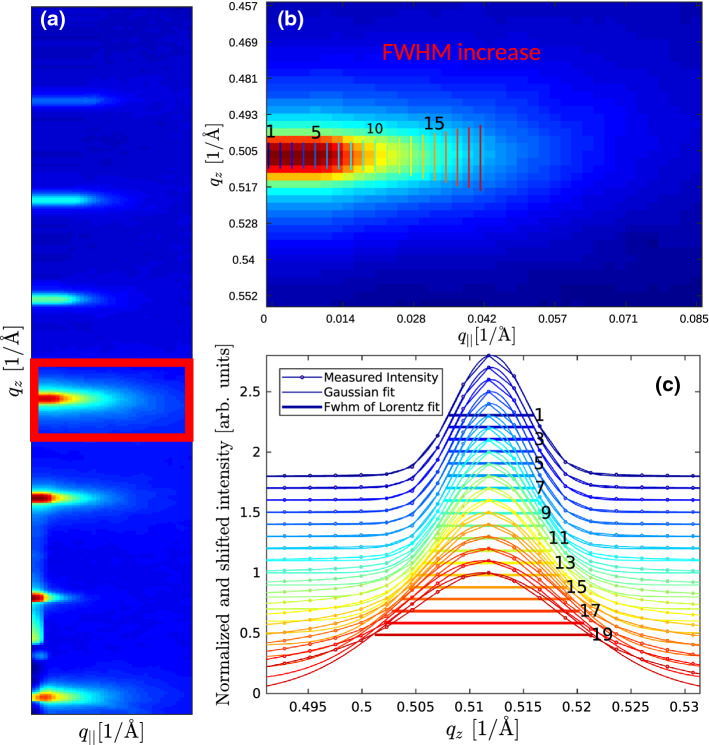
Illustration of the line profiles and the measured FWHM as a function of $q_\parallel$, for the example of the fourth ($n=4$) Bragg sheet at 70 $\%$. **a** The GISAXS pattern, with highlighted ROI around the fourth Bragg sheet. **b** Enlarged ROI ($n=4$) with illustration of the FWHM ($q_z$) and increase along $q_\parallel$. **c** The intensity line profiles as a function of $q_z$, and the corresponding FWHM indicated by horizontal bars, as obtained from least-square fits (increasing $q_{||}$ from top to bottom). For the display, each profile was normalized to its maximum value, and shifted for clarity by $-0.1$ for each increment in $q_{\parallel }$. The total range covered by this exemplary case was from $q_{\parallel }= 0 \mathring{A}^{-1}$ to q$_{\parallel }= 0.044\mathring{A}^{-1}$

Figure 5 illustrates the analysis of Bragg sheet line profiles. For each Bragg sheet order *n*, a respective rectangular region-of-interest (ROI) was selected; see the green frame in **a**, exemplified for $n=4$. The ROI is enlarged in **a**, with schematically indicated vertical white lines, along which the profiles were extracted and fitted. Profiles are shown in **c** after normalization and vertical shift for clarity. The expected increase in FWHM as a function of $q_\parallel$ is clearly evident. It simply reflects the fact that bilayer undulations with shorter wavelength are less correlated vertically than fluctuations of large length scale (short spatial frequencies). This behavior is analogous to conformal roughness in inorganic multilayer systems, but here derived from the fluctuations in thermal equilibrium which are governed by the smectic free energy density (Hamiltonian).

**Fig. 6:**
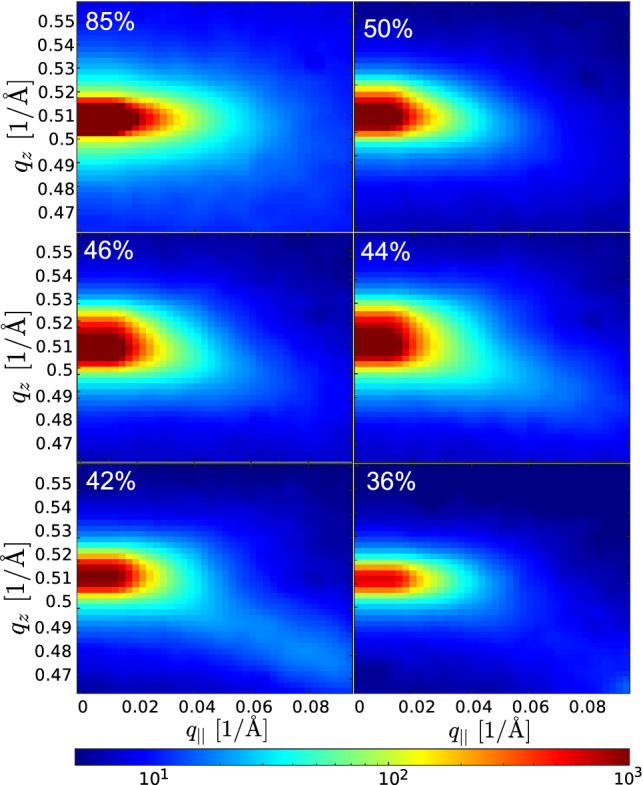
Bragg sheet intensity distribution and width (logarithmic color scale), for selected RH, illustrating the broadening in the pre-critical regime. The fourth Bragg sheet is shown for 85 $\%$, 50 $\%$, 46 $\%$, 44 $\%$, 42 $\%$ and 36 $\%$ RH. The observed changes are quantified by the FWHM obtained from Gaussian fits and plotted in Fig. 7

Next, Fig. 6 illustrates the RH dependence. The fourth Bragg sheet is plotted for different RH values (logarithmic colorbar) to illustrate the lineshape changes in the pre-critical regime, before presenting further analysis below. The selected ROI covers a range $\varDelta q_z = 0.1\,\mathring{\text {A}}\,^{-1}$ centered around the maximum and a range $0\le q_{\parallel } \le 0.095\,\mathring{\text {A}}\,^{-1}$ in the out-of-plane direction. The width increase in the pre-critical regime can already be observed by eye before any fit. As we detail below, this indicates a reduction in the vertical correlation length of thermal fluctuations, hence an increase in $\lambda$ or, respectively, a softening of the compressional modulus. This width increase is accompanied by an increase of diffuse scattering at the lower right of the ROI towards the location of the neighboring peak (0,1,11), emerging in the rhombohedral phase when stalks crystallize.

**Fig. 7:**
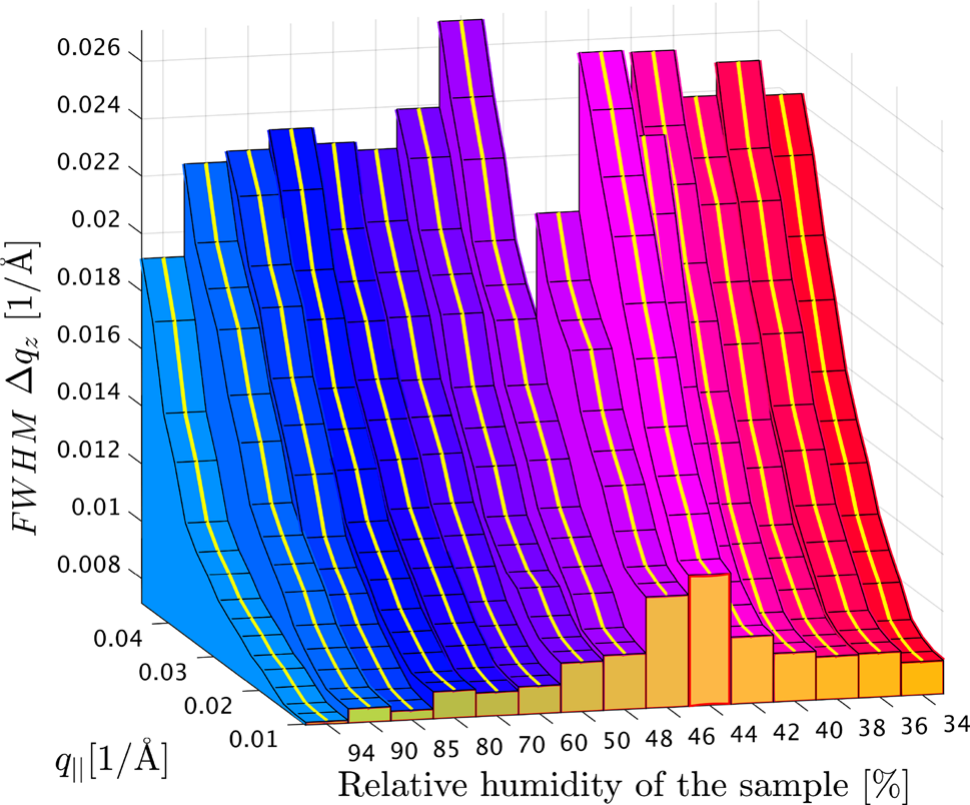
Results of the Bragg sheet width analysis. The FWHM along $q_z$ is plotted for the example of the fourth Bragg sheet as a function of $q_\parallel$ for all measured RH. The widths of the Bragg sheet close to $q_z$-axis is highlighted in yellow. Note that the baseline of the plotted FWHM is 72 $\cdot$ $10^{-3}$ Å$^{-1}$, corresponding to the minimum FWHM at 94 $\%$. The width and the quadratic coefficient of the FWHM profile increase during the pre-critical regime, just above the transition from the lamellar to the rhombohedral phase

Figure 7 presents the FWHM along $q_z$ as a function of $q_\parallel$ for all RH measured in a waterfall plot, again for the example of the fourth Bragg-sheet. For all RH, the characteristic increase with $q_\parallel$ is observed, which is predicted to be quadratic according to linear smectic elasticity theory. An anomalous behavior with elevated FWHM values is observed in the pre-critical regime (purple). In fact the minimum FWHM increases from 72 $\cdot$ $10^{-3}\,\mathring{\text {A}}\,^{-1}$ at $RH=94$ $\%$ to 117 $\cdot$ $10^{-3}\,\mathring{\text {A}}\,^{-1}$ at $RH=46$ $\%$, corresponding to an increase by more than 50 $\%$. All curves are shown for the range of $q_\parallel$ selected for fitting to the quadratic law, with $\lambda$ as a free fit parameter. The exploitable range is limited towards high $q_\parallel$ by the background and low signal-to-noise ratio, and at low $q_\parallel$ by the scattering geometry. Note that $q_\parallel <10^{-3}\,\mathring{\text {A}}\,^{-1}$ cannot be reached for $n=4$ at the given $\alpha _i$, and is subject to interpolation/regridding artifacts when transforming from detector pixels to $(q_\parallel ,q_z)$.

### de Gennes length

**Fig. 8:**
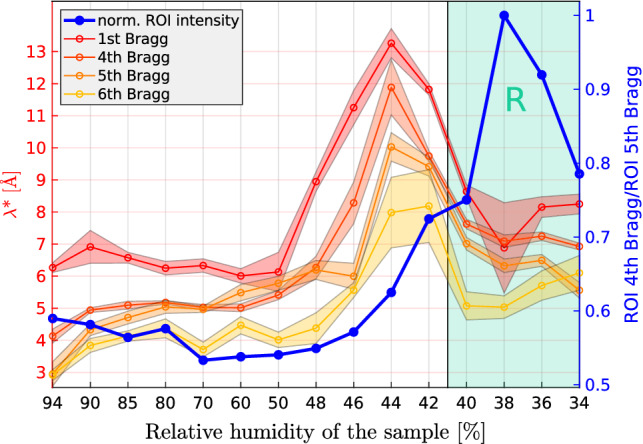
RH dependence of the de Gennes length. The effective de Gennes length $\lambda _n^*$ obtained from the FWHM analysis using Eq. 6, for the Bragg sheets $n=1,4,5,6$, along with the normalized ROI intensity quantifying the off-axis off-specular scattering and its transformation into the peak of the rhombohedral lattice upon stalk crystallization. The rhombohedral phase is highlighted by the semi-transparent green color. The de Gennes parameter $\lambda$* shows a maximum in the pre-critical regime and declines when stalks have crystalized in the rhombohedral phase. The errors shown correspond to the root-mean-square error of the fit. The normalized off-specular scattering intensity (blue line) is also plotted as function of RH, showing an increase in the pre-critical regime before it peaks reflecting the formation of a rhombohedral lattice peak

Next, we present the de Gennes lengths $\lambda$ extracted from the $\varDelta q_z(q_\parallel )$ curves. As described above in Sect.  2, each order *n* is fitted independently, in terms of an effective value $\lambda ^*_n$. Figure 8 shows the results for $n=1,4,5,6$, i.e., all Bragg sheets which have a sufficient signal. Note that the intensities of the $n=2$ and $n=3$ sheets are too low, in particular around RH$\simeq 70 \%$, due to a form factor minimum, reflecting the bilayer electron density profile of DOPC Yamamoto et al. (2015). For all analyzed Bragg sheets ($n=1,4,5,6$). The functional form of RH dependence of the effective coefficients $\lambda ^*_n (RH)$ is identical. In particular, the curves all peak in the pre-critical regime. The values decrease systematically with *n*, which is a result of the non-linear integral relating the diffuse structure factor to the corresponding correlation functions (Salditt 2005). From the systematic shifts, however, we can infer that the $n=1$ values approximate the true $\lambda$ fairly well. Accordingly, the de Gennes parameter increases from ($6.1 \pm 0.6$) Å at 50 $\%$ RH to a maximum value of ($13.3 \pm 0.5$) Å at 44 $\%$ for the first Bragg sheet, and declines again in the rhombohedral phase to ($8.6 \pm 0.2)\,\mathring{\text {A}}$ at 40 $\%$ RH. In addition, Fig.  7 also displays the change in off-specular scattering intensity (blue curve, right axis), in order to quantify the pre-critical diffuse scattering at the transition to the rhombohedral phase. To obtain this curve, the scattering was integrated in a ROI near the 4th Bragg sheet, as indicated in Fig.  10, divided by the total scattering intensity of a background ROI, and finally normalized to its maximum value. Reflecting the strong rhombohedral reflection with Miller indices (0,1,11), the intensity curve peaks in the rhombohedral phase, but also shows a pronounced increase and shoulder in the pre-critical regime.

### Bending and compression modulus

We now address the dependence of the bending rigidity $\kappa$ and the compression modulus *B* on relative humidity *RH*, as computed from $\eta$ (from specular reflectivity), and $\lambda$ (from non-specular reflectivity/diffuse scattering around Bragg sheets). Here, $\kappa$ depends on *RH* through $K=\kappa /d$ as well as *d*(*RH*), which is easily extracted from the Bragg sheet position. Interpolation is used where *RH*-values of specular (home laboratory) and non-specular (synchrotron) data did not exactly coincide.

**Fig. 9:**
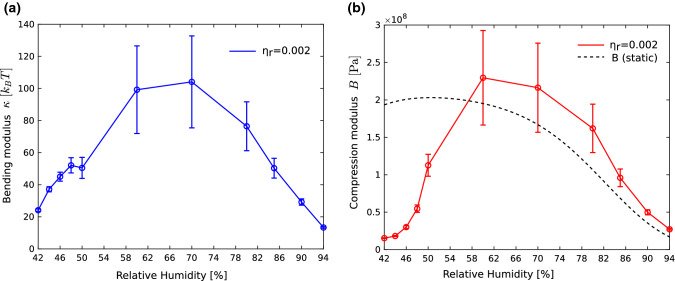
**a** The bending rigidity $\kappa$ and **b** the compression modulus *B* as a function of *RH*. The error bars reflect the error interval in the reference value $\eta _r$, i.e., the Caillé parameter at $RH_r=65\%$. Note that the moduli describe the response to thermal fluctuations, and hence relate to dynamic bending and compressional modes. For this reason *B* differs from the static disjoining pressure obtained from the osmotic pressure technique, plotted for comparison (dotted line), after polynomial fitting of previously reported DOPC data (Aeffner 2011; Aeffner et al. 2012). The main new finding here is the softening in the pre-critical regime, above the phase transition to the rhombohedral phase. In this regime, transient stalks result in a reduction of the (effective) moduli of bending and compression. In particular, *B* shows a significant decrease around 44 % RH before stalks crystallize in the rhombohedral phase

Figure 9 presents the resulting curves for (a) $\kappa$ and (b) *B*. As is well known from the literature, the moduli increase, when moving from full to partial hydration, as the system gets stiffer when inter-bilayer water is removed. The main new result here is the decrease of the moduli, and in particular the compression modulus at the pre-critical regime. With respect to the highest relative humidity measured $RH=94\%$, *B* decreases to $55\%$, and with respect to the intermediate range even to $6\%$! At the same time, the bending rigidity evaluates to $\kappa =13.3 k_BT$ at $RH=94\%$, and after reaching its maximum of around $100 k_BT$ in the intermediate range, falls down again to $\kappa =24 k_BT$. Comparison to the literature values is difficult, since these differ significantly between techniques (Bochicchio and Monticelli 2016; Dimova 2014). For single-component phosphatidylcholine bilayers, values range from (5.9 ± 1.2) k$_B$T (POPC) (Kummrow and Helfrich 1991) to (38.5 ± 0.8) k$_B$T (Henriksen et al. 2006) from micropipette aspiration. From X-ray lineshape analysis, $\kappa =24 k_BT$ was previously reported for DOPC (Nagle et al. 2015), similarly $\kappa =23 k_BT$ for DMPC (fluid phase) (Mennicke et al. 2006). The values of *B* at high RH are also in the range of typical literature values of single-component phosphatidylcholine bilayers, compared for example to $B=46$ MPa reported in Schneck et al. (2008) and Schneck (2010), for DPPC at $T=60$ $^{\circ }$C and 95 $\%$ RH.

Of course, as explained above, we also have to keep in mind that the curves can only be computed after fixing one free parameter, notably $\eta _r=0.002$ at the reference value $RH_r=65\%$. This parameter was chosen in view of a plausible fluctuation amplitude $\sigma$ and plausible value $\kappa$. The error interval of $\eta _r$ was estimated by min/max values of $\eta _{r,min}=0.0015$ and $\eta _{r,max}=0.0025$, respectively, which then set the error bars of for $\kappa (RH)$ and *B*(*RH*). As one can see, the ’free’ parameter $\eta _r$ only influences the intermediate regime, while the DW-factor at high *RH* and towards the phase transition is sufficiently high, such that the choice of $\eta _r$ has very little influence. In particular, the softening in the pre-critical regime in the vicinity of the phase transition, which is the main result of this work, is not affected.

Finally, we stress again that $\eta$ could in principle also be determined without free parameter for example from lineshape exponents, as well as from full q-range fits of the diffuse scattering. Since $\eta$ is proportional to the mean square fluctuation amplitude $\sigma ^2$ of the bilayers, it has a significant influence on scattering tails and the power law decay of the specular peaks along $q_z$ (Rawicz et al. 2000; Nagle and Tristram-Nagle 2000). However, in partially hydrated systems on solid support, the power-law tails of the specular Bragg peaks are not observed (Salditt et al. 1999) if $\eta$ is too weak, and the decay of the Bragg peak intensity with *n* becomes a better suited observable. In practice, lineshape exponents are not easily extractable from reflectivity for small $\eta \lessapprox 0.02$, when large wavelength undulations are suppressed by finite size and boundary conditions of aligned stacks. A full analysis of the structure factor with proper boundary conditions is still possible based on the Bragg sheet intensity decay with $q_\parallel$ but extremely involved (Constantin et al. 2003). Here we decided against such analysis since it is not only numerically more involved, but also less robust than the $\lambda$-analysis and $\eta$-analysis, as presented above. Apart from these challenges associated with structure factor calculations and lineshape fitting, we also believe that linear smectic elasticity theory at these mesoscopic length scales should always be regarded as an effective theory, and the resulting elasticity parameters should be interpreted primarily with regard to their relative dependencies.

## Interpretation and conclusion

For the interpretation, we have to consider the two main experimental findings in the pre-critical regime: (A) the increased diffuse scattering around the position which later becomes a prominent peak of the stalk phase, and (B) the anomalous elasticity behavior with a pronounced softening in the pre-critical regime, characterized by an decrease of the modulus *B* as well as of the bending rigidity $\kappa$. We first interpret the diffuse scattering signal (A) which appears in a range of $q_\parallel$ corresponding to lateral length scales close to the bilayer thickness. Given the high $\lambda$-values, it cannot be explained by membrane dynamics or rearrangements on mesoscopic lengths, such as bilayer undulations. Further, due to the absence of sharp off-axis peaks, any long-range crystalline symmetry must be excluded. At the same time, the pronounced modulation with respect to both $q_\parallel$ and $q_z$ is indicative of short-range order. In a single-component lipid system such as DOPC, and also in view of the phase transition, the only convincing explanation is a liquid phase of correlated stalks, probably associated with transient formation of a stalk and its decay back to flat bilayers. The fact that the signal is peaked also along $q_z$ suggests that the formation of a first stalk may lower the threshold of forming further stalks in its vicinity, i.e., we must put forward the idea that stalks form (and decay) in a correlated manner.

According to the second main finding (B), the pre-critical regime is also characterized by an anomalous elasticity behavior of the membrane stack, i.e., a decrease in inter-bilayer compressional modulus and bending rigidity, see in particular the pronounced cusp in the *B*(*RH*) curve. This indicates a strong decrease of the free energy associated with the compression of the multilamellar system. Upon a fluctuation resulting in closer spacing of of adjacent bilayers within a certain region of the stack, the restoring force is significantly softened. This can be explained by a local formation of short-range ordered stalks, which favor smaller water layers and can better encompass such inter-membrane distance fluctuations which would otherwise be suppressed by hydration repulsion. According to Aeffner et al. (2012), we can expect that this happens as soon as the local headgroup–headgroup distance reaches the critical value of $d_{\text {w}}=(9.0\pm 0.5)\,\mathring{\text {A}}$. The present results suggest that this can also happen transiently without crystallization, further lowering the free energy by the entropic gain resulting from fluctuating stalk numbers and positions.

**Fig. 10:**
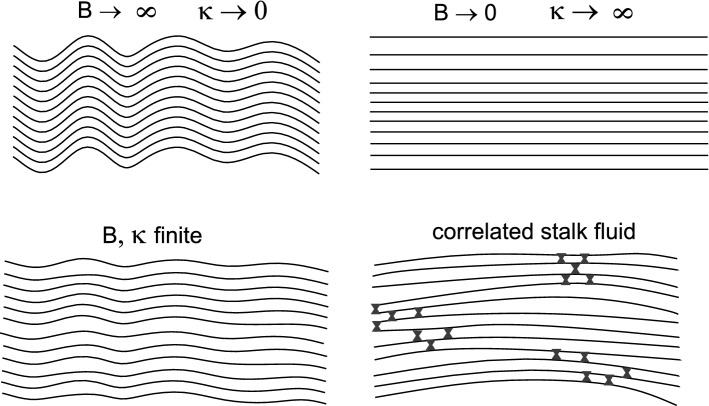
Schematic of thermal fluctuations in a multilamellar lipid system with smectic liquid-crystalline elasticity. For $B\rightarrow \infty$ positional fluctuations in one membrane can only be relaxed by bending of the entire stack (upper left, conformal undulations), since the inter-bilayer distance $d_w$ cannot be varied from its equilibrium value. For $\kappa \rightarrow \infty$ positional fluctuations persist throughout the entire membrane area (upper right), since curvature is prohibited. Only compressional (breathing) modes are allowed. For finite *B* and $\kappa$, the response to a local shift in membrane position is given by a superposition of bending and compressional modes (lower left). A fluid of correlated stalks exists in the pre-critical regime. Stalks form collectively in patches characterized by local compression (lower right). This coupling of membrane compression and stalk formation results in an anomalous increase in compressibility

In summary, we have observed and described an interesting phase state of soft matter, formed by a smectic lipid system hosting a correlated fluid of stalks. This stalk fluid forms in the lamellar phase just above the phase transition to the rhombohedral phase, where stalks crystallize. In contrast to normal fluids, the constituents of this fluid, i.e., the stalks, are not conserved in number, and form transiently in a dynamic manner. As a next step, observations of the associated time scales, for example by X-ray photon correlation spectroscopy or by neutron spin echo spectroscopy would certainly be very useful to confirm this interpretation and to reveal the corresponding time scales.

Finally, we may also ask about the biological relevance of this effect. When two membranes come closer, driven by fusiogenic proteins, and the patches are sufficiently large such as in cell–cell fusion or larger exocytotic vesicles, it seems not unplausible that collective formation of stalks with mutual elastic interaction could play a role. While elastic interaction could result in a reduction of enthalpy per stalk, transient formation and relaxation of stalk structures would increase entropy, both acting to reduce the free energy barrier along the fusion pathway. While several transient stalks would hence form and decay again, only a single stalk and hemifusion diaphragm may ultimately develop into a fusion pore. This speculation that collective stalk formation may also occur in a system of only two adjacent bilayers could eventually be tested by quantifying lipid mixing rates in hemifusion events of large unilamellar vesicles.

## Figures and Tables

**Fig. 11:**
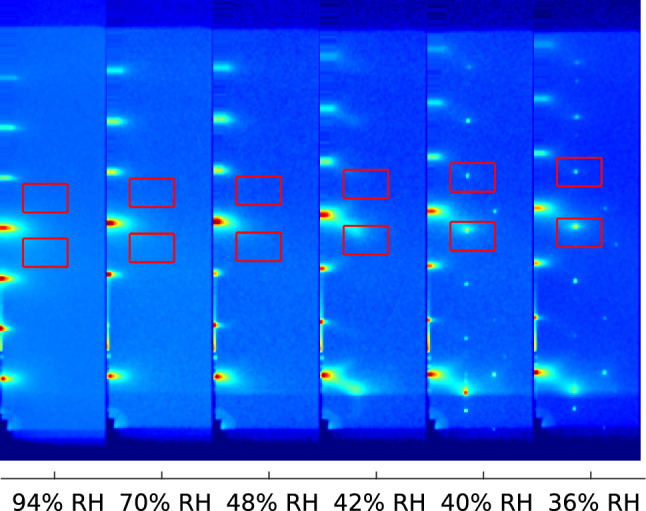
Selection of ROI for off-specular scattering, illustrated for GISAXS patterns at different RH after transformation to $\mathbf{q}$. ROIs are indicated by red rectangles. The main signal ROI is selected around the location where the (0,1,11) peak forms in the rhombohedral phase. The ROI location shifts with RH, to account for the shifting lamellar periodicity *d*. The signal ROI is divided by a reference ROI without pre-critical diffuse scattering (upper red rectangle)

**Fig. 12:**
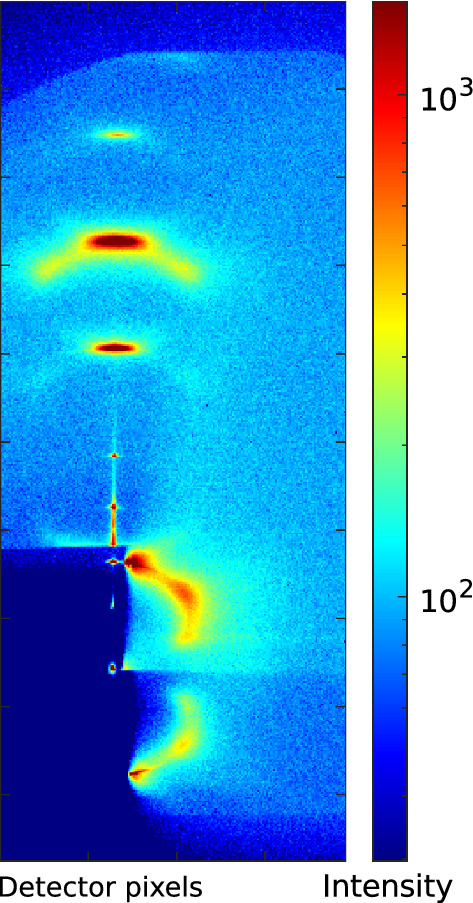
GISAXS pattern in the pre-critical regime with beamstop moved to the side, such that the scattering below the sample horizon at $\alpha _f<0$ becomes visible. For the figures of the main text, the scattering below the sample horizon at negative exit angle $\alpha _f<0$ was blocked by the beamstop in Figs. 3 and 10. When the beamstop is moved to the side (while still blocking the primary beam), the GISAXS pattern to negative $\alpha _f$ becomes visible since the silicon wafer with the membrane stack is still partially transparent at 25 keV

**Fig. 13:**
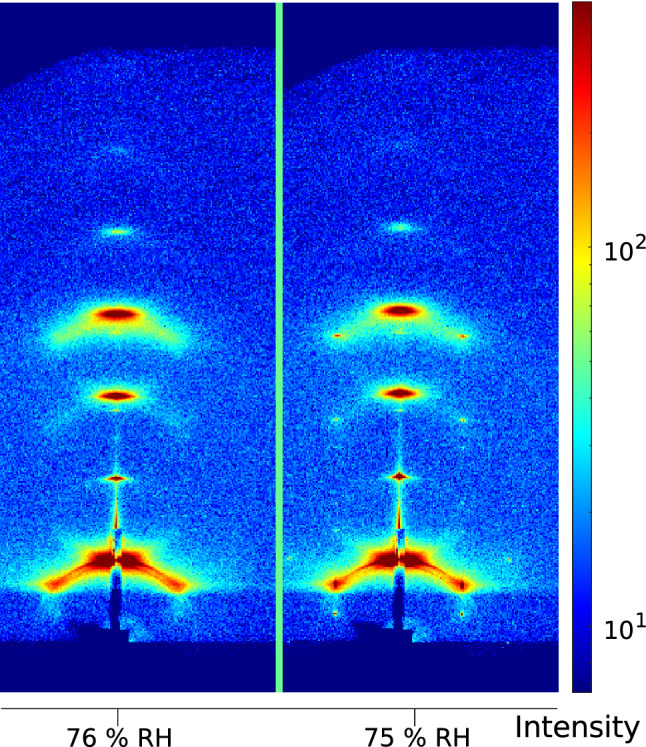
The pre-critical diffuse scattering with the characteristic pattern is also observed for DOPC:DOPE (1:1), where the transition shifts to higher RH

**Fig. 14:**
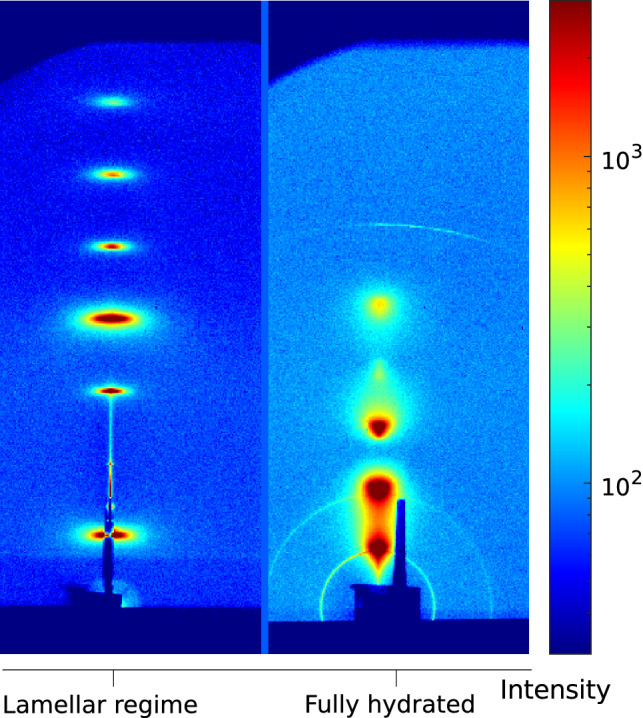
Comparison of the GISAXS pattern of a DOPC lipid stack at (left) partial and (right) full hydration, where the sample is immersed in ultra-pure water. The peak lineshape reflects the higher excitation of thermal fluctuations and the corresponding change of the structure factor

## Appendix: additional datasets

Additional datasets are provided, to illustrate (a) how the ROIs were selected for the off-specular intensity curve as a function of RH (Fig. 11); (b) how the pre-critical GISAXS scattering extends below the sample horizon (Fig. 12); (c) that the pre-critical behavior also appears for other lipid composition (Fig. 13), and finally (d) how the GISAXS pattern changes from partial to full hydration (Fig. 14).
